# Cerebral Microbleeds in the Patients With Acute Stroke Symptoms

**DOI:** 10.3389/fneur.2018.00988

**Published:** 2018-11-21

**Authors:** Sung Hyuk Heo, Dongwhane Lee, Yong Chul Kwon, Bum Joon Kim, Kyung Mi Lee, Cheryl D. Bushnell, Dae-IL Chang

**Affiliations:** ^1^Department of Neurology, Kyung Hee University Hospital, Seoul, South Korea; ^2^Department of Radiology, Kyung Hee University Hospital, Seoul, South Korea; ^3^Department of Neurology, Wake Forest School of Medicine, Winston-Salem, NC, United States

**Keywords:** cerebral microbleeds, diffusion weighted image, stroke, transient ischemic attack, magnetic resonance image, stroke

## Abstract

**Background:** Some patients with acute stroke symptoms do not show hyperintensities on diffusion-weighted image (DWI). A few case reports have indicated that acutely developed cerebral microbleeds (CMBs) might cause focal symptoms. This study sought to investigate the incidence and characteristics of symptomatic CMBs in the patients with acute stroke symptoms but without DWI ischemic lesions.

**Methods:** We enrolled the patients with acute stroke symptoms who underwent magnetic resonance imaging including DWI and gradient echo (GRE) sequences within 7 days after symptom onset, at our prospective stroke registry. We then identified patients without DWI-positive ischemic lesions but with CMBs in the relevant brain regions.

**Results:** Between January 2005 and February 2012, we identified 235 DWI-negative transient ischemic attack (*n* = 221) and stroke (*n* = 14) patients from 2129 consecutive patients at our registry. In total, 16 patients had CMBs corresponding to the focal symptoms. Among these 16 patients, 12 patients showed a hyperintense rim on DWI around a microbleed suspected to be related to focal symptoms; of the 12 patients, 7 experienced stroke symptoms for more than 24 h. However, the symptoms in the remaining patients (5 patients with the hyperintense rim and 4 patients without the hyperintense rim) improved within 24 h.

**Conclusion:** Symptomatic microbleeds are infrequent but not rare in the patients with acute stroke symptoms. Perihematomal edema around an acute CMB can cause a hyperintense rim on DWI. Our results suggest that a combination of DWI and GRE imaging can help diagnose acute symptomatic CMBs.

## Introduction

Diffusion-weighted imaging (DWI) has been considered the most sensitive imaging technique for detection of acute ischemic stroke. However, some patients with acute stroke symptoms do not show any high signal intensities suggesting early infarction on DWI (1). These patients are diagnosed as having stroke mimics, such as seizure, migraine, or transient ischemic attack (TIA), if their symptoms are relieved within 24 h. Patients with some types of stroke, particularly with mild small vessel stroke in the posterior circulation, can be DWI-negative within the first few days after stroke. However, when the symptoms persist for more than 24 hours and no ischemic lesions are found on follow-up images, both patients and neurologists are frustrated in such situations (2).

A few case reports have documented the acute development of cerebral microbleeds (CMBs), which might be expected to cause focal symptoms (3–6). In addition, the hyperintense rim on DWI may provide a clue to an acutely developed lesion (5, 6). This study aimed to evaluate how many DWI-negative stroke or TIA patients have relevant CMB lesions, and the imaging and clinical characteristics of these patients with different symptom durations were also examined.

## Methods

### Subjects and study design

From January 2005 to February 2012, we consecutively recruited patients, who had acute stroke symptoms and underwent magnetic resonance imaging (MRI) including DWI and gradient echo (GRE) sequences within 7 days after symptom onset at the prospective ischemic stroke registry of our hospital. CMBs were defined as small, rounded, well-defined hypointense lesions within the brain parenchyma with clear margins, ranging from 2 to 10 mm in size on GRE images. We identified the patients with symptomatic CMBs if the patients did not have any DWI-positive ischemic lesions but had CMBs in the relevant brain regions. Subsequently, we analyzed the MR findings (size, location, and DWI changes) and clinical features of these patients. We investigated whether the hyperintense rim on DWI was present around symptomatic CMBs. TIA was defined as a brief episode of neurological dysfunction, caused by focal brain or retinal ischemia, with clinical symptoms lasting < 24 h (7). This study was approved by an independent Ethics Committee at Kyung Hee University Medical Center (KMC IRB 1221-02).

### Magnetic resonance imaging protocol

MRI was performed on a 1.5T MR system (Genesis Signa; GE Healthcare, Milwaukee, Wisconsin). All patients were scanned in the supine position in the MR scanner and their heads were placed within an 8-channel phased array neurovascular coil. The MRI protocol included GRE, DWI, T1-weighted image, T2-weighted image, fluid attenuation inversion recovery imaging, and time of flight MR angiography. GRE sequences were acquired in the axial plane using the following parameters: repetition time, 500 ms; echo time, 15 ms; flip angle, 20°; slice thickness, 5 mm; slice gap, 1.5 mm; field of view, 220 mm; matrix size, 256 × 160–192; and number of excitations, 2. Axial DWI sequences were obtained using the following parameters: repetition time, 6,300 ms; echo time, 70 ms; flip angle, 90°; slice thickness, 5 mm; slice gap, 1.5 mm; field of view, 220 mm; matrix size, 128 × 128; and b, 0/1,000 s/mm^2^. Apparent diffusion coefficient (ADC) maps were constructed automatically.

Two independent neurologists (D.L. and Y.C.K.) investigated CMBs in the DWI-negative stroke or TIA patients. If the raters disagreed on the presence or absence of CMBs, discrepancies were resolved by a consensus meeting.

### Statistical analysis

Baseline demographic data are expressed as mean ± standard deviation or median (interquartile range) for continuous variables or as frequencies for categorical variables. Significant differences between experimental groups and controls were assessed using analysis of variance, chi-square test, Fisher's exact test, and Kruskal-Wallis test. *P* < 0.05 were considered statistically significant. All statistical analyses were conducted using the SPSS 22.0 package for Windows (SPSS, Chicago, IL, United States).

## Results

We identified 235 (11.0%) DWI-negative patients with acute stroke symptoms from 2,129 consecutive patients at our registry. TIA was diagnosed in 221 patients (94.0%) and stroke in 14 patients (6.0%) according to symptom duration. Among these patients, 16 patients (6.8%) had CMBs in the brain regions corresponding to symptoms (Table 1), and 39 patients had asymptomatic CMBs. Symptomatic CMBs were more common in men than in women, and asymptomatic CMBs were more common in patients with the history of previous stroke or the previous use of antithrombotics. The patients with symptomatic and asymptomatic CMBs were older and had higher scores in modified Fazekas scale and poorer scores in the clinical scales than those without CMBs. One patient (0.4%) had focal cortical superficial siderosis in DWI-negative patients with acute stroke symptoms, and the patient was in the group with asymptomatic CMBs.

**Table 1:** Baseline demographic and clinical characteristics.

	**No CMB (*n* = 180)**	**Asymptomatic CMB (*n* = 39)**	**Symptomatic CMB (*n* = 16)**	***P*-value**
Men, n (%)	102 (56.7)	20 (51.3)	14 (87.5)	0.038
Age	61.3 ± 12.6	66.5 ± 9.7	65.7 ± 9.8	0.030^*^
Previous stroke, n (%)	20 (11.1)	20 (51.3)	4 (25.0)	< 0.001
Previous antithrombotics, n (%)	19 (10.6)	12 (30.8)	2 (12.5)	0.004
Hypertension, n (%)	99 (55.0)	27 (69.2)	11 (68.8)	0.179
Diabetes, n (%)	37 (20.6)	9 (23.1)	4 (25.0)	0.876
Dyslipidemia, n (%)	73 (40.6)	14 (35.9)	6 (37.5)	0.851
Current smoker, n (%)	39 (21.7)	6 (15.4)	4 (25.0)	0.623
Atrial fibrillation, n (%)	2 (1.1)	2 (5.1)	0 (0.0)	0.234
BMI (kg/m^2^)	24.3 ± 3.1	23.6 ± 2.8	24.0 ± 3.0	0.393
Glucose (mg/dl)	114.9 ± 50.7	111.5 ± 30.7	108.6 ± 27.4	0.833
HbA1c (%)	5.9 ± 1.0	6.0 ± 0.8	5.8 ± 0.8	0.714
Total cholesterol (mg/dl)	178.9 ± 38.8	170.6 ± 42.3	177.9 ± 41.7	0.494^*^
HDL cholesterol (mg/dl)	45.3 ± 12.6	43.8 ± 12.9	42.8 ± 11.6	0.633^*^
TG cholesterol (mg/dl)	127.7 ± 80.2	114.8 ± 64.2	116.4 ± 59.6	0.593^*^
Systolic BP (mmHg)	143.7 ± 24.7	142.6 ± 27.6	143.3 ± 25.9	0.970^*^
Diastolic BP (mmHg)	85.9 ± 10.6	85.4 ± 11.4	81.5 ± 11.6	0.306^*^
Modified Fazekas scale (median, IQR)	1 (0,1)	1 (1,2)	1 (1,2)	< 0.001^‡^
ABCD2 score (median, IQR)	4 (3,5)	5 (4, 6)	4 (4, 5)	0.003^‡^
Length of stay (median, IQR), days	3 (2,5)	4 (3, 6)	4 (2, 5)	0.051^‡^
Admission NIHSS (median, IQR)	0 (0, 2)	2 (0, 4)	2.5 (1, 4)	< 0.001^‡^
Discharge NIHSS (median, IQR)	0 (0, 0)	0 (0, 2)	0 (0, 2.5)	< 0.001^‡^
Discharge mRS (median, IQR)	0 (0, 0)	1 (0, 1)	1 (0, 1)	< 0.001^‡^

*P-values are for Chi-squared test unless indicated*.

* *P-value is for ANOVA*.

*^†^P-value is for Fisher's exact test*.

‡ *P-value is for Kruskal-Wallis test*.

*BMI, body mass index; HbA1c, hemoglobin A1c; HDL, high-density cholesterol; TG, triglyceride; BP, blood pressure; IQR, interquartile range; NIHSS, NIH stroke scale; mRS, modified Rankin scale*.

**Figure 1:**
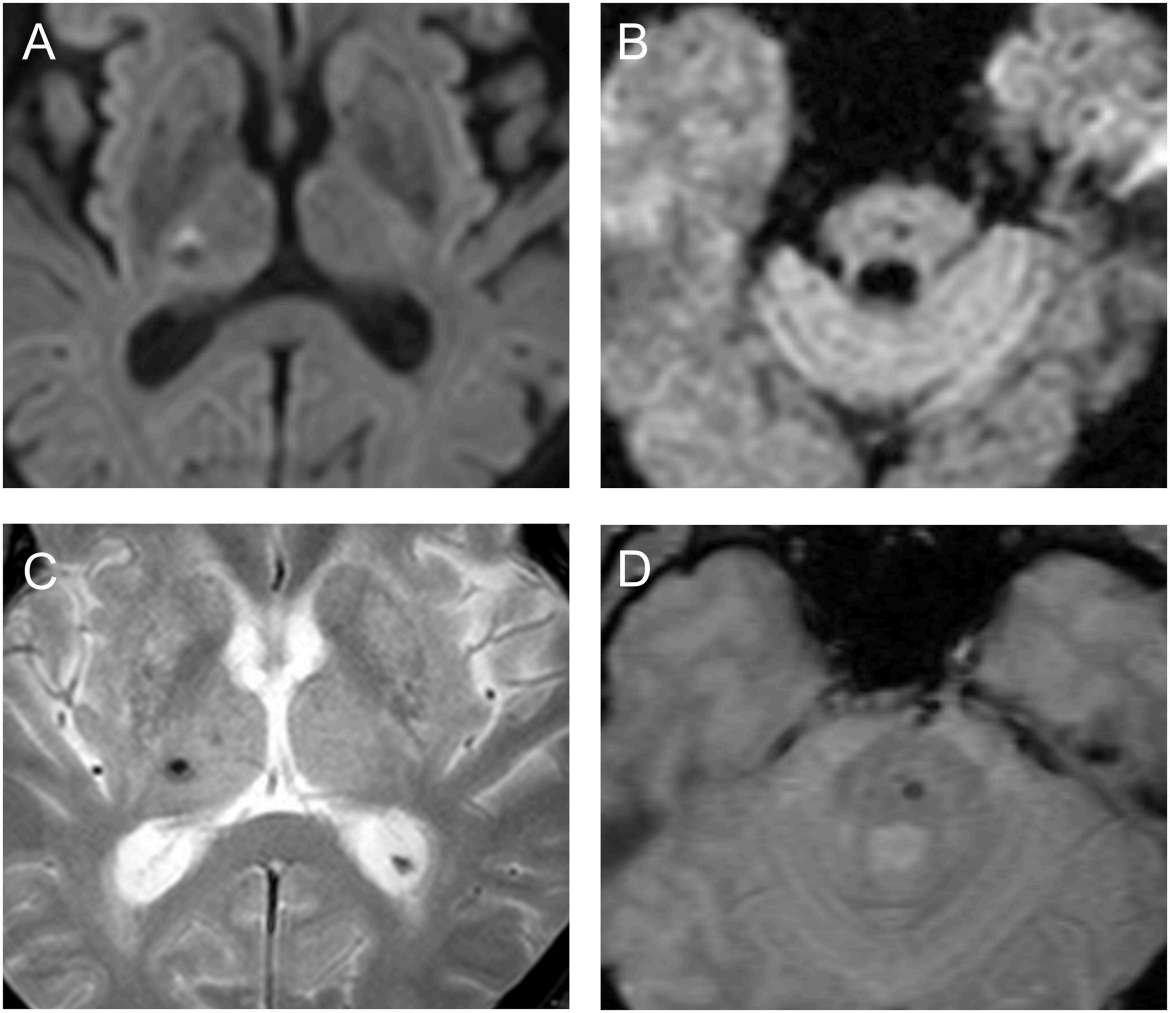
Diffusion-weighted images **(A,B)** and gradient-echo sequences **(C,D)** show suspicious symptomatic cerebral microbleeds with (patient 1, **A,C**) or without (patient 13, **B,D**) the hyperintense rim in the patients.

The clinical manifestations of most patients (13/16) were lacunar syndromes, but two patients had cerebellar ataxia and one had diplopia. In total, 12 patients (75%) showed a hyperintense rim on DWI around a microbleed suspected to be related to the symptoms (Table 2 and Figure 1); among the 12 patients, 7 patients, accounting for half of all imaging-negative stroke patients (*n* = 14), claimed that their stroke symptoms had lasted more than 24 h. However, all 4 patients without the hyperintense rim had improved symptoms within 24 h.

**Table 2:** Summary of acute stroke symptoms and cerebral microbleeds in the relevant areas in 16 patients.

**Patient**	**Sex**	**Age range**	**Risk factors**	**Stroke syndrome**	**Duration**	**ABCD2 score**	**Lesion**	**Size (mm)**	**Hyperintense rim**	**ADC of the hyperintense rim (10^−3^ mm^2^/s)**	**CSF (mm^2^/s)**	**Previous stroke history**	**Total CMB count (lobar/deep)**
1	M	66–70	HT, DM, HL	Pure sensory	> 24 h	5	Thalamus	4	(+)	0.47 ± 0.05	1.53	(+)	9 (1/8)
2	M	76–80	HT	Ataxic hemiparesis	< 24 h	4	pons	3	(+)	0.39 ± 0.03	0.94	(+)	5 (0/5)
3	M	76–80	HT, AF, smoking	Dysarthria	> 24 h	4	Subcortex	7	(+)	1.05 ± 0.12	3.12	(+)	10 (5/5)
4	M	71–75	HT, DM, HL	Pure motor	> 24 h	7	Subcortex	7	(+)	0.46 ± 0.03	1.53	(–)	3 (2/1)
5	M	56–60	HT, HL, smoking	Pure motor	< 24 h	4	Internal capsule	2	(+)	0.39 ± 0.04	1.45	(–)	1 (0/1)
6	M	56–60	Smoking	Pure sensory	> 24 h	4	Pons	2	(+)	0.38 ± 0.08	1.55	(–)	5 (0/5)
7	M	71–75	HT, HL	Dysarthria, ataxia	< 24 h	4	Cerebellum	8	(+)	0.89 ± 0.50	2.66	(–)	2 (2/0)
8	M	66–70	HT	Dysarthria, facial palsy	> 24 h	3	Subcortex	2	(+)	0.77 ± 0.11	3.09	(+)	2 (0/2)
9	M	71–75	HL, smoking	Pure motor	< 24 h	6	Subcortex	5	(+)	0.42 ± 0.03	1.57	(+)	3 (1/2)
10	F	61–65	None	Dysarthria	< 24 h	4	Subcortex	5	(+)	0.81 ± 0.10	2.85	(–)	1 (0/1)
11	M	51–55	HT, DM, HL, smoking	Pure motor	> 24 h	6	Pons	4	(+)	0.76 ± 0.12	3.06	(–)	4 (1/3)
12	M	71–75	HT, HL	Ataxic hemiparesis	> 24 h	4	Thalamus	8	(+)	0.42 ± 0.04	1.49	(+)	13 (3/10)
13	M	51–55	HT	Pure motor	< 24 h	5	Pons	3	(–)			(+)	3 (0/3)
14	M	61–65	HT, DM	Ataxia	< 24 h	5	Cerebellum	3	(–)			(–)	1 (1/0)
15	M	41–45	HT, HL	Pure motor	< 24 h	4	Thalamus	3	(–)			(–)	1 (0/1)
16	F	66–70	HT	Diplopia	< 24 h	3	Midbrain	2	(–)			(–)	1 (0/1)

*ADC, apparent deficiency coefficient; CSF, cerebrospinal fluid; CMB, cerebral microbleed; M, male; F, female; HT, hypertension; DM, diabetes mellitus; HL, hyperlipidemia; AF, atrial fibrillation*.

## Discussion

With the development of brain imaging techniques, the diagnosis of acute stroke is no longer difficult; in recent years, it is possible to diagnose an imaging-positive ischemic stroke even if no symptoms are observed. However, some patients do not have any DWI abnormalities despite their acute symptoms. CMBs, generally considered asymptomatic, have been found to be a diagnostic cause of stroke and TIA symptoms (3–6). However, although these CMBs may be observed in suspected stroke or TIA patients, it is difficult to establish a direct association between CMBs and acute stroke symptoms without the brain images immediately before the symptom onset.

The hyperintense rim is frequently observed on DWI in patients with acute intracranial hematoma and is thought to be due to perihematomal edema (8, 9). Two studies suggested that a combination of DWI and GRE can help identify acute symptomatic CMBs (5, 6). The presence of a hyperintense rim on DWI, as well as the presence of CMB identified by GRE, may be a better indication of symptomatic CMBs. In this study, we objectively calculated the ADC values. This study found that CMBs with the hyperintense rim were observed in 7 out of 14 DWI-negative stroke patients. Therefore, some cases of imaging-negative stroke might be caused by symptomatic CMBs in the clinical setting. However, it is difficult to ascertain whether the CMB causes the stroke syndrome in the 4 patients without the hyperintense rim. This might be because CMBs without the hyperintense rim are too small (≤ 3 mm in size) to cause perihematomal edema or because these CMBs are asymptomatic or are not related to this event (10).

Transient focal neurological episodes have been found to relate to cerebral amyloid angiopathy and cortical superficial siderosis (11, 12). Only one patient in the group with asymptomatic CMBs had cortical superficial siderosis, and no perilesional hyperintense rim on DWI was observed in the patient. In addition, none of the symptomatic CMBs was located in the cerebral cortex in the patients. Therefore, cortical superficial siderosis might be unrelated to the symptoms of the patients in the present study.

The stroke subtype of most patients were lacunar syndromes. It is thought that the stroke location causing symptoms by a small lesion is likely to be a subtype of small vessel occlusion. Little or no neurological sequelae were found in the patients with symptomatic CMBs. However, the patients with CMBs had slightly higher scores of admission and discharge NIH stroke scale and discharge modified Rankin scale, and the results might be caused by older age, more frequent history of previous stroke, and the burden of small vessel disease.

Our study has several limitations. First, the patients underwent 1.5T MRI in this study, although the detection rate of CMBs and superficial siderosis may increase by using 3T MRI and/or susceptibility-weighted imaging. Second, our study was a retrospective observational study. The prevalence of symptomatic CMBs may be different in a prospective study. Therefore, caution is required regarding accepting our results as conclusive evidence. Third, only 6.8% of our patients had been suspected as having symptomatic CMB lesions, and the evidence supporting causal relationship between these CMBs and acute stroke symptoms was weak. However, symptomatic CMBs were found in half of imaging-negative stroke patients whose symptom lasted more than 24 h, and to the best of our knowledge, this is the first study investigating the association of CMBs and imaging-negative TIA or stroke.

In conclusion, DWI, in conjunction with GRE imaging, is useful in diagnosing not only acute ischemic stroke but also acute symptomatic CMBs by identifying the hyperintense rim, and a significant number of imaging-negative stroke patients may have symptomatic CMBs.

## Author contributions

SH and D-IC developed the concept of this study. SH, DL, YK, BK, and KL acquired and analyzed data. SH, KL, and CB drafted and revised the manuscript, figures, and tables.

### Conflict of interest statement

The authors declare that the research was conducted in the absence of any commercial or financial relationships that could be construed as a potential conflict of interest.
